# Endoscopic management of gastric ectopic pancreas with repeated ulcerations and bleeding: A case report

**DOI:** 10.1002/deo2.338

**Published:** 2024-01-27

**Authors:** Tomoya Kimura, Yohei Minato, Susumu Banjoya, Toshifumi Iida, Koichi Furuta, Shinya Nagae, Yohei Ito, Hiroshi Yamazaki, Nao Takeuchi, Shunya Takayanagi, Yoshiaki Kimoto, Yuki Kano, Takashi Sakuno, Kohei Ono, Sakiko Miura, Teppei Morikawa, Ken Ohata

**Affiliations:** ^1^ Department of Gastrointestinal Endoscopy NTT Medical Center Tokyo Tokyo Japan; ^2^ Department of Diagnostic Pathology NTT Medical Center Tokyo Tokyo Japan

**Keywords:** bleeding, ectopic pancreas, endoscopic resection, gastroscopy, submucosal tumor

## Abstract

A 25‐year‐old man was referred to our center for investigation of a gastric submucosal tumor and an ulcer that had developed on its oral side. Endoscopic ultrasonography findings suggested the presence of an ectopic pancreas, and treatment with an oral proton pump inhibitor was planned for the ulcer. Over the subsequent 3 years, the patient endured recurring epigastric pain and episodes of passing black stools. Emergency endoscopy revealed that the morphology of the gastric submucosal tumor had transformed into a pedunculated polyp‐like morphology with a bleeding ulcer at the apex of the lesion. Endoscopic hemostasis using hemostatic forceps was performed. However, the patient continued to pass black stools. In light of the persistent symptoms and unique morphology of the lesion, endoscopic resection was attempted as a curative approach. The lesion was excised by hot snare polypectomy. Post‐treatment, the patient exhibited no signs of recurrence, marking a successful resolution. Three months later, a gastroduodenal endoscopy showed that the excised site had undergone scar formation without recurrence of the lesion. This case holds significant clinical value as it demonstrates the efficacy of a minimally invasive treatment strategy in managing repeated bleeding ulcerations of an ectopic pancreas, ultimately achieving a complete cure.

## INTRODUCTION

An ectopic or heterotopic pancreas is defined as a pancreatic tissue located outside the normal pancreatic parenchyma in an aberrant location with vascular and nerve supplies independent of the main supplies of the pancreas. Ectopic pancreatic tissues are frequently incidentally detected in gastrointestinal tract evaluations. Although bleeding from an ectopic pancreas has been reported in various gastrointestinal locations, such as the stomach, duodenum, jejunum, and Meckel's diverticulum,^1^, ^2^, ^3^, ^4^ endoscopic treatment remains rare.^5^ Herein, we report an intricate case of a gastric ectopic pancreas, which was characterized by recurrent ulcerations and bleeding.

### Case report

**FIGURE 1:**
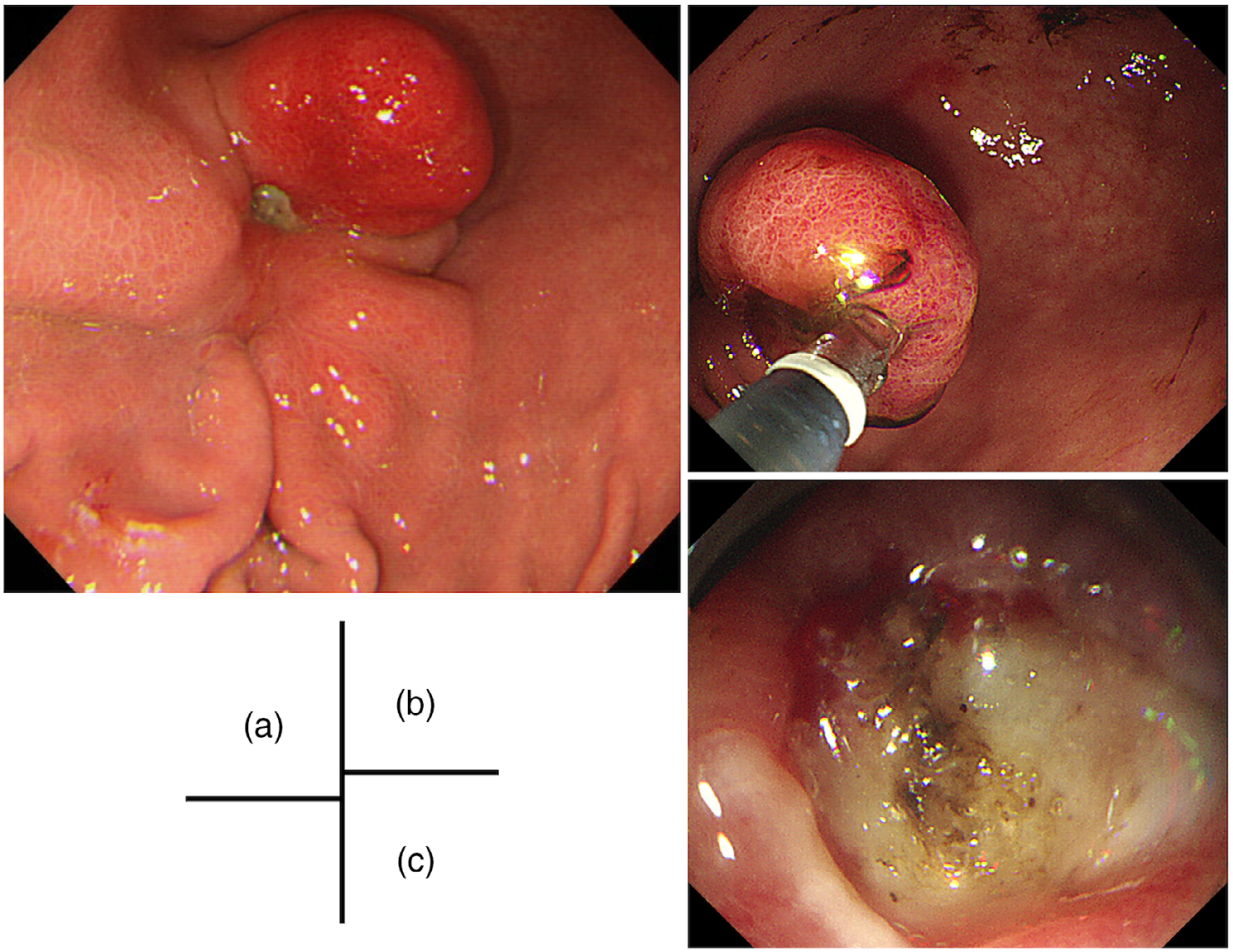
Endoscopic findings of the lesion Initial findings and during the endoscopic hemostasis. (a) A 20‐mm submucosal lesion was detected in the greater curvature of the gastric angulus which was associated with an ulcer that had developed at the basal end. (b) The lesion had changed to a pedunculated polyp‐like morphology, with the ulcerated at the apex of the polyp‐like morphology. (c) Endoscopic hemostasis was performed for bleeding at the exposed blood vessels on the ulcerated surface.

**FIGURE 2:**
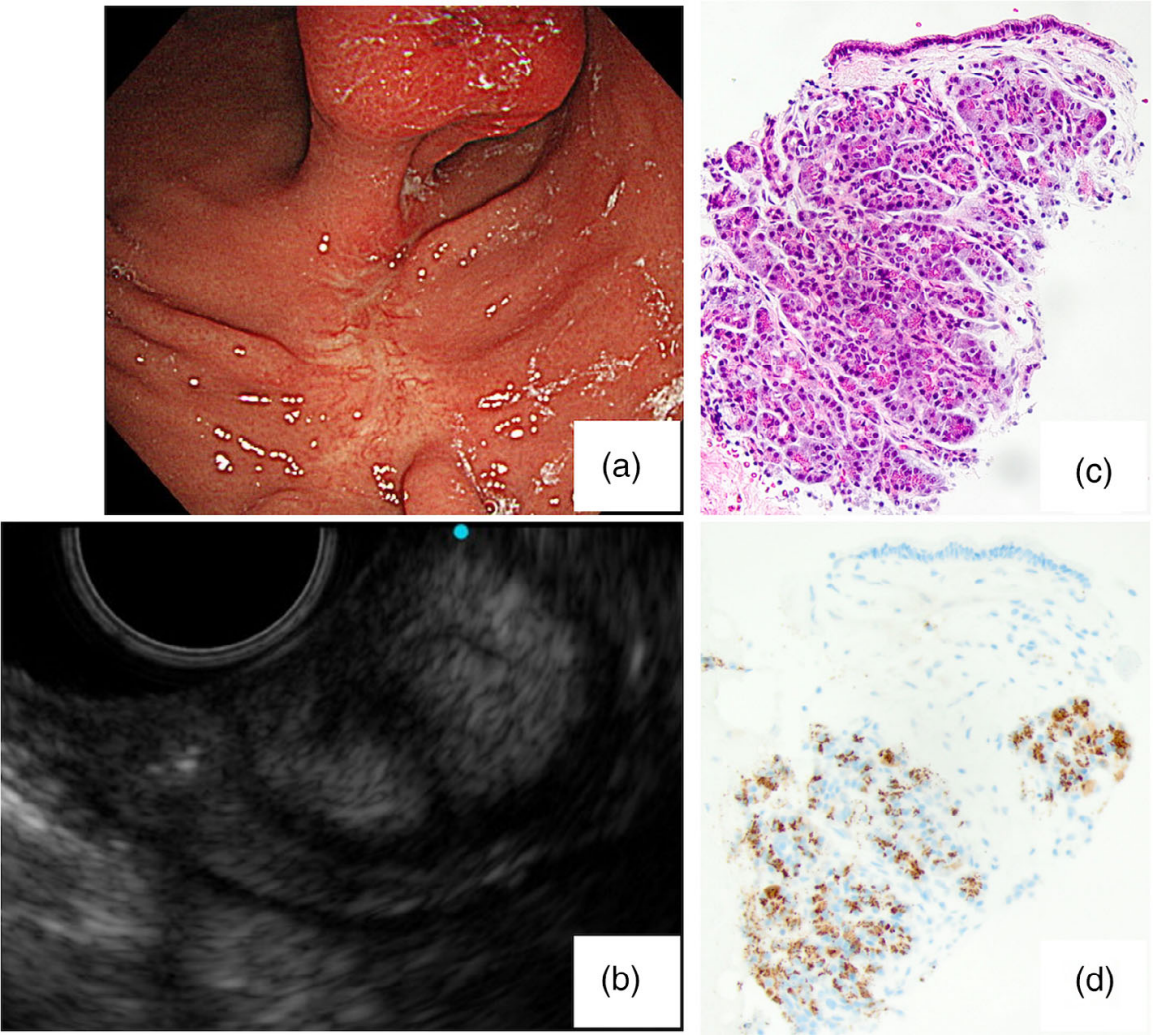
Endoscopic findings after 3 years. (a) The lesion had enlarged to 25 mm, the stalk was elongated, and the ulcer at the basal end of the lesion was scarred. (b) Endoscopic ultrasonography (20 MHz frequency) showed a hyperechoic lesion mainly in the submucosal layer of the gastric wall. (c) The pathological findings of the endoscopic ultrasonography‐fine‐needle aspiration biopsy. Pancreatic acinar cell (H&E staining, ×200). (d) The acinar cells were positive for trypsin (Trypsin staining, ×200). The Histopathological diagnosis was ectopic pancreas.

**FIGURE 3:**
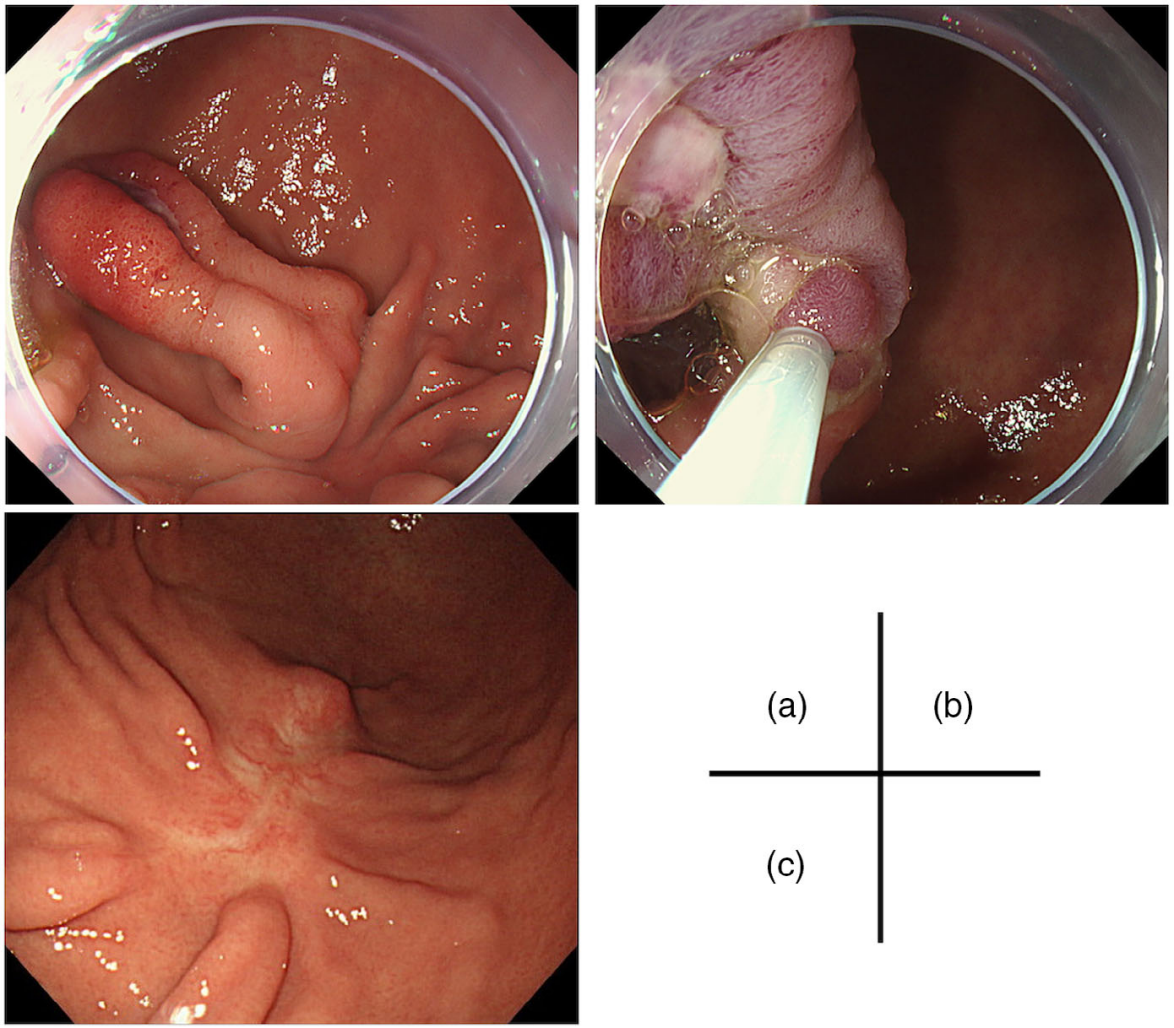
Images during the endoscopic excision and after 3 months. (a) The morphology of the lesion transformed as the stalk elongated, and the ulcer localized at the apex of the polyp‐like lesion. (b) The lesion was en block excised by hot snare polypectomy. (c) The excised site had undergone scar formation with no recurrence of the lesion.

**FIGURE 4:**
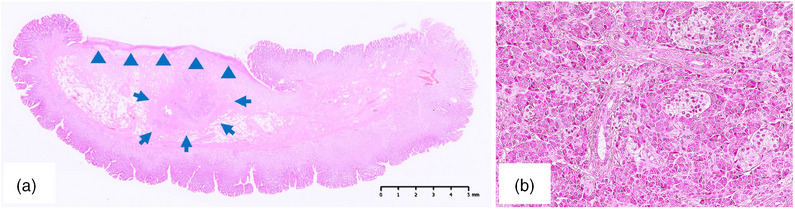
Histopathological findings of ectopic pancreatic. (a) Ectopic pancreas tissue was located in the submucosal layer (arrow; hematoxylin and eosin [H&E] staining, bar = 5 mm). The apex of the lesion forms an ulcer (arrowhead). (b) The ectopic pancreas consisted of pancreatic acini, ducts, and islets of Langerhans. (H&E staining, ×200).

A 25‐year‐old man initially underwent a gastroduodenal endoscopy (GS) after presenting with epigastric pain. The GS revealed the presence of a reddish SMT lesion in the greater curvature of the gastric angulus, which was associated with an ulcer that had developed at the basal end. (Figure 1a). The patient was referred to our hospital for further investigations. Endoscopic ultrasonography was performed, revealing a heterogeneous hypoechoic tumor. The presence of foci and ductal structures, characteristic of an ectopic pancreas, was observed, leading us to consider it an ectopic pancreas. The ulcer responded well to oral proton pump inhibitor therapy with resolution of the presenting symptoms. The patient underwent continued follow‐up for evaluation of the lesion. However, the patient was lost to follow‐up after the subjective improvement of his symptoms. In the course of the attrition, the epigastric pain recurred intermittently. He underwent self‐medication with over‐the‐counter medicines. Three years after his last visit to our hospital, he presented again with symptoms of worsening epigastric pain, nausea, and black stools. Laboratory analyses revealed a red blood cell count of 2.80 × 10^6^/μL, hemoglobin level of 8.8 g/dL, hematocrit of 25.6%, serum amylase level of 151 IU/L, and C‐reactive protein concentration of 0.03 mg/dL. Testing for serum anti‐*Helicobacter pylori* immunoglobulin G antibodies yielded a negative result (<3.0 U/mL). GS revealed that the SMT had changed from an elevated morphology to a pedunculated polyp‐like morphology, with the ulcerated portion now situated at the apex of the polyp‐like morphology. The previously observed ulcer had undergone scar, and the SMT size had clearly increased. Hemostatic forceps were used to secure hemostasis of the exposed blood vessels on the ulcerated surface endoscopically (Figure 1b,c). After 1 month, an endoscopic ultrasonography‐fine‐needle aspiration biopsy confirmed the presence of pancreatic acinar cells, leading to a definitive diagnosis of an ectopic pancreas (Figure 2). The lesion had enlarged with associated symptoms of bleeding. Therefore, an endoscopic treatment was planned. However, he stopped visiting the hospital again. One year later, he presented to our hospital due to symptom recurrence and decided to undergo endoscopic treatment. The endoscopic excision was performed using upper gastrointestinal endoscopes (GIF‐Q260J; Olympus). Due to the elongation of the stalk of the lesion and the localization of the ulcer at the apex of the polyp‐like lesion, a hot snare polypectomy was deemed to be a suitable intervention. The lesion was excised as far as possible from the base of the polyp‐like morphology after hemostasis with a detachable snare (Endoloop; Olympus; Figure 3a,b). Hot snare polypectomy was performed with a high‐frequency snare (Dualoop; Medicos Hirata) and a VIO300D electrical unit (Erbe) in Endo Cut mode Q (effect 3, 45 W). The size of the excised specimen was 28×15×12 mm. Histopathological evaluation revealed that the lesion was located in the submucosal layer and consisted of pancreatic tissues, including acini, ducts, and islets of Langerhans (Figure 4). In the ulcerated area of the polyp‐like lesion, infiltration of intense inflammatory cells was observed. Furthermore, in the stroma of the polyps, increased proliferation of adipose tissue, collagen fibers, and smooth muscle tissue, suggesting the presence of chronic inflammation. Three months later, a GS showed that the excised site had undergone scar formation with no recurrence of the lesion (Figure 3c). The patient has since not experienced a recurrence of the symptoms during the 2‐year follow‐up period.

## DISCUSSION

In this case, we observed the morphological changes of an ectopic pancreas over a 4‐year period and excised the lesion by endoscopic treatment. Since the ectopic pancreas repeatedly underwent ulcerations and caused anemia, endoscopic treatment was indicated. A hot snare polypectomy was selected as the curative treatment because of the lesion's unique morphology. After the excision of the lesion, a curative treatment was achieved, as no recurrence of the lesion or symptoms was observed. Several reports of surgical resection as a curative treatment for bleeding from the ectopic pancreas are available in the literature.^1^, ^3^, ^4^ However, the choice of endoscopic treatment has rarely been made in such cases.^5^

An ectopic pancreas is typically asymptomatic. However, it can cause a range of symptoms depending on its location, size, and secretions. Pancreatic cells of an ectopic pancreas have exocrine and endocrine functions similar to those of normal pancreatic cells. Pancreatitis, pancreatic pseudocyst formation, gastric outlet obstruction, gastric ulcer bleeding, and malignant transformation^6^ have been associated with cases of ectopic pancreas reported in the literature.^7^ Among patients with symptomatic ectopic pancreas, the most commonly reported symptom is abdominal pain. Dyspepsia, nausea, heartburn, diarrhea, weight loss, hematemesis, and melena are described infrequently. In this case, the initial symptom of epigastric pain emerged approximately 1 year prior to the hospital visit. Subsequently, with the enlargement of the lesion, the patient experienced increasing epigastric discomfort, nausea, and passage of black stools.

Endoscopically, ectopic pancreases are typically observed as elevated, broad‐based submucosal lesions with umbilicated appearances. This central umbilication is due to the subjacent pancreatic duct.^8^ In this case, no findings of umbilication were present, and the entire lesion had elevated papillary‐like morphology at the time of the initial endoscopy. To the best of our knowledge, no reports of a gastric ectopic pancreas undergoing transformation into a pedunculated polyp‐like morphology, as was observed in this case.

The etiology of the bleeding in this patient with a heterotopic pancreas was considered to be related to ulcerations of the gastric mucosa covering the abnormal lesion. Chemical irritation and inflammation can result in the breakdown of the mucosal defense, and pancreatic enzymes, such as elastase, may contribute to the weakening and erosion of the underlying blood vessel walls.^9^ Moreover, persistent inflammation resulting from gastric ectopic lesions induces swelling and congestion within the submucosal layer of the stomach. This congestion within the fragile submucosal blood vessels can result in bleeding and subsequent release into the gastric lumen. In this case, the initial upper abdominal pain was thought to be due to ulcerations of the ectopic pancreas, considering the aforementioned factors. Additionally, the serum amylase level was mildly elevated. Therefore, the involvement of pancreatitis cannot be ruled out. Subsequently, after the observation of changes in the polyp‐like morphology due to gastric peristalsis, the bleeding was considered to emanate from the apex of the lesion due to traction ischemia and mechanical stimulation by peristalsis.

With respect to the treatment of ectopic pancreas, asymptomatic cases are typically managed by follow‐up observation. However, as in this case, a lesion that bleeds repeatedly needs to be treated. An ectopic pancreas is typically a benign lesion that requires minimally invasive treatment. However, ectopic pancreases are usually located in the submucosal‐to‐muscle layer. Surgical resection is often the curative treatment, whereas endoscopic treatment is seldom chosen.^10^ In this case, endoscopic excision was feasible because of the lesion's unique morphology and preoperative endoscopic ultrasonography evaluation findings, which indicated that the main location of the ectopic pancreas was within the submucosal layer of the polyp‐like morphology. Therefore, the patient underwent curative treatment by hot snare polypectomy.

In conclusion, a patient with a gastric ectopic pancreatic lesion with associated repeated bleeding underwent curative treatment by hot snare polypectomy. After the excision of the lesion, no recurrence of the symptoms has been reported during the 2‐year follow‐up period.

## CONFLICT OF INTEREST STATEMENT

None.

## ETHICS STATEMENT

All procedures followed have been performed in accordance with the ethical standards laid down Declaration of Helsinki and its later amendments.
